# To What Extent and under What Conditions Is the Collar-Crown a Cause of Pericemental Inflammation

**Published:** 1894-04

**Authors:** C. L. Goddard

**Affiliations:** San Francisco.


THE
AMERICAN JOURNAL
—OF—
DENTAL SCIENCE.

VOL. XXVII. BALTIMORE, APRIL, 1894. NO. 12.

ARTICLE I.

### TO WHAT EXTENT AND UNDER WHAT CONDITIONS IS THE COLLAR-CROWN A CAUSE OF PERICEMENTAL INFLAMMATION.

BY PROF. C. L. GODDARD, A. M., D. D. S., SAN FRANCISCO.

Read before the San Francisco Dental Association.

The extent of the inflammation caused by the collar-crown varies from none at all to sufficient to cause absorption of the walls of the socket and loss of root. We can see cases in all stages between these two. The first will be marked by a slight redness at the gum margin *not* accompanied by pain. A stage further advanced will be shown by increased redness and swelling of the gum margins, and tenderness of the tooth to percussion. We use the term "tenderness of the tooth," from habit, when we mean tenderness of the peridental membrane, from compression between the root and the walls of the alveolus. This condition may be accompanied by a slight extending of the tooth from the socket.

The next stage will be an aggravated continuation in which, perhaps, the whole peridental membrane is involved, and the tooth more noticeably pushed out of its socket by the membrane thickened in the process of inflammation. In this stage the tenderness may be so great as to prevent use of the tooth and even of the other teeth adjoining by preventing their occlusion.

In these stages the amount of tenderness may depend on the shape of the root. If the root has parallel sides for most of its length, and the inflammation extends no farther than the sides are parallel, there will be no pain produced by force brought to bear in a direct line with the long axis of the tooth, but only by a force exerted laterally. In this condition the tooth may still be very useful.

If, however, the root is cone shaped—that is, if it tapers all the way from the gum margin to the apex—it is clear that any force applied in the line of the long axis of the root will compress the inflamed peridental membrane, and cause pain much greater than that caused by lateral force, because the muscles of mastication enable greater force to be brought to bear in that direction. The last stages of the wasting away of the socket and consequent extreme looseness of the tooth, will be accompanied by more or less complete loss of usefulness from the tooth moving so much as to fail to masticate, even if there were not sufficient tenderness to prevent its use. This wasting away of the walls of the alveolus may continue till the peridental membrane has entirely disappeared and the tooth or root drops for want of attachment.

Still further, in answer to the question, To what extent will inflammation be caused by the collar-crown? we may say that if the collar-crown is properly fitted, it will cause no more inflammation than a well-fitted crown without a collar, and not nearly so much as a collarless crown badly fitted, for manifestly if a collarless crown be fitted so that it projects on any or all sides of a root—that is, if the base of a crown be larger than the root, and these projecting edges be left sharp and angular—it will cause inflammation; or, if the base of a crown is smaller than the base of a root, so that the root projects laterally, its sharp corners or edges will naturally cause inflammation of the gum tissue which may grow over them. This inflammation may extend to the peridental membrane, or, if the root and base of the crown be of the same size, yet imperfectly fitted to each other so that gum tissue will project between, it will be inflamed by the sharp edges over which it extends; or, if this space be filled with cement, gutta-percha or amalgam, which is allowed to protrude from the point, this will cause inflammation.

"Under what condition is the collar-crown a cause of pericemental inflammation?" This is simply answered by saying, by misfit;" for we have misfit collars as well as misfit clothing. As most roots are conical in shape, and the collar must be pushed on over the large end, it is evident that the further a collar is pushed the greater will be the misfit, the more the collar will impinge on surrounding tissues, and the greater will be the inflammation. If the collar-crown be a whole gold crown slipped over the contour sides of a natural crown, it will be much larger than the root at the cervical margin, and will not fit at all, but will impinge upon the gum tissue, in the endeavor to push it under the gum line. The collar itself will irritate, and the root will be filled with food particles or other deleterious substances with mucus or saliva, which, degenerating or decomposing, will aid in causing inflammation.

Secondly, even if the sides of the root or root and crown be parallel, either by nature or by trimming, and the band be extended too far under the gum line, it will itself impinge upon the peridental membrane and cause inflammation. This may ensue from not adapting or trimming the upper edge of the collar to the contour of the gum line, and, in the endeavor to fit the collar, just under the edge of the lingual, buccal or labial portion of the gum, the proximal portion of the collar may be pushed up too far, as in normal conditions the alveolar process and peridental membrane are more prominent or extend nearer the end of the root between the teeth than on the other surfaces.

Thirdly, even if the band is reasonably well fitted upon a root or tooth, already slightly loose, its constant movement on the tissues during use of the tooth may cause inflammation. A tooth or root may be slightly loose and yet be sufficiently firm to warrant its being crowned, in which case the collar may be extended only to and not under, the gum line; or, in case of a gold crown, may stop considerably short of the gum line and yet be so perfectly fitted and its edges so nicely beveled as not to be a cause of injury.

You will notice that our subject, in the form of a question, does not inquire for a remedy; yet if it did, our only answer would be, to fit the collar properly; which means, to fit it so as not to cause inflammation. The fit may not be perfect, for it is not always possible to make a perfect fit, and yet, in many cases, no injury will be done. For Atkinson told us long ago that there is no tissue of the human body that will stand so much abuse as the peridental membrane.—*Pacific Coast Dentist.*

